# Supplementary material for: 2,4-Thiazolidinedione in Well-Fed Lactating Dairy Goats: I. Effect on Adiposity and Milk Fat Synthesis
Source: Vet Sci. 2019 May 17;6(2):45. doi: 10.3390/vetsci6020045 (PMC6632146; doi:10.3390/vetsci6020045)
Supplement: Supplementary file 1 [file vetsci-06-00045-s001.zip › vetsci-484037-supplementary/Table S1.docx]

**Table S1**. Nutrient Composition of the forages used during the experimental period.

| Chemical analysis |  | Alfalfa hay | Orchard Grass hay |
| --- | --- | --- | --- |
| Dry Matter (DM) | % | 89.7 | 91.4 |
| NE_L_ (Mcal/Lb) | % DM | 0.69 | 0.53 |
| Crude Protein (CP) | % DM | 23.2 | 12.2 |
| Adjusted crude protein | % DM | 22.6 | 12.2 |
| Soluble Protein | % CP | 46.0 | 26.0 |
| ADF | % DM | 25.7 | 37.0 |
| aNDF | % DM | 33.2 | 60.0 |
| NFC | % DM | 27.3 | 18.6 |
| TDN | % DM | 65.0 | 59.0 |
| Calcium | % DM | 0.97 | 0.31 |
| Phosphorus | % DM | 0.24 | 0.20 |
| Magnesium | % DM | 0.51 | 0.24 |
| Potassium | % DM | 2.64 | 2.28 |
| Sodium | % DM | 0.39 | 0.05 |
| Sulfur | % DM | 0.34 | 0.20 |
| Iron | ppm | 1460 | 223 |
| Zinc | ppm | 20 | 18 |
| Copper | ppm | 8 | 8 |
| Manganese | ppm | 48 | 112 |
| Molybdenum | ppm | 5 | 0.4 |
