# Supplementary material for: 2,4-Thiazolidinedione in Well-Fed Lactating Dairy Goats: I. Effect on Adiposity and Milk Fat Synthesis
Source: Vet Sci. 2019 May 17;6(2):45. doi: 10.3390/vetsci6020045 (PMC6632146; doi:10.3390/vetsci6020045)
Supplement: Supplementary file 1 [file vetsci-06-00045-s001.zip › vetsci-484037-supplementary/Table S2.docx]

**Table S2**. Rations fed to *Saanen* lactating goats during the experiment period based on body weight (BW) and milk initial milk yield.

| ***Goats features*** |  | | | | | | |
| --- | --- | --- | --- | --- | --- | --- | --- |
| BW (kg) | 60 | 70 | 70 | 80 | 80 | 90 | 90 |
| Milk yield (kg/d) | 2.1-3.2 | 2.1-3.2 | 3.5-4.8 | 2.1-3.2 | 3.5-4.8 | 2.1-3.2 | 3.5-4.8 |
| ***Feed components*** | ***Daily Feed (kg head^-1^, DM basis)*** | | | | | | |
| Alfalfa Hay | 0.5 | 0.5 | 1.2 | 0.5 | 1.5 | 0.6 | 1.0 |
| Orchardgrass Hay | 1.6 | 1.8 | 1.2 | 2.2 | 2.0 | 2.4 | 2.8 |
| Kountry Buffet | 0.5 | 0.6 | 0.5 | 0.5 | 0.5 | 0.5 | 0.5 |
| SweetLix Minerals (g/d) | 14.2 | 14.2 | 14.2 | 14.2 | 14.2 | 14.2 | 14.2 |
