# Supplementary material for: 2,4-Thiazolidinedione in Well-Fed Lactating Dairy Goats: I. Effect on Adiposity and Milk Fat Synthesis
Source: Vet Sci. 2019 May 17;6(2):45. doi: 10.3390/vetsci6020045 (PMC6632146; doi:10.3390/vetsci6020045)
Supplement: Supplementary file 1 [file vetsci-06-00045-s001.zip › vetsci-484037-supplementary/Table S3.docx]

**Table S3**. Composition of vitamin drench (amount per 10 mL solution) given to each goat daily.

| Component | Daily g per goat | Vitamin | IU supl.^4^ |
| --- | --- | --- | --- |
| Vitamin D (CHS^1^) | 0. 2 | A | 17,000 |
| Vitamin E (CHS^2^) | 12.5 | D | 7,000 |
| Vitamins and Electrolytes mix (Durvet^3^) | 7.5 | E | 1,700 |

^1^Contains 12,000 IU/Kg (CHS, MN, USA)

^2^Contains 227,000 IU/Kg (CHS, MN, USA)

^3^Contains a maximum of 37% of sodium, and a minimum of 3% of potassium, 2,267,965 IU/kg of Vitamin A, 907,186 IU/kg of Vitamin D3, 907 IU/kg of Vitamin E, 2 mg/kg of Vitamin B12, 907 mg/kg of menadione, 680 mg/kg of riboflavin, 1,134 mg/kg of d-pantothenic acid, and 227 mg/kg of thiamine hydrochloride, 2,268 mg/kg of niacin, 3,402 mg/kg of ascorbic acid, 227 mg/kg of pyridoxine hydrochloride and 59 mg/kg of folic acid (Durvet, MO, USA).

^4^IU/day supplemented by the drench
