# Supplementary material for: 2,4-Thiazolidinedione in Well-Fed Lactating Dairy Goats: I. Effect on Adiposity and Milk Fat Synthesis
Source: Vet Sci. 2019 May 17;6(2):45. doi: 10.3390/vetsci6020045 (PMC6632146; doi:10.3390/vetsci6020045)
Supplement: Supplementary file 1 [file vetsci-06-00045-s001.zip › vetsci-484037-supplementary/Table S4.docx]

**Table S4**. Primer-pairs newly designed for the present experiment

| **Symbol** | **NCBI Acc.** | **Start** | **Primer** | **Amp size** | **Amplicon sequence** |
| --- | --- | --- | --- | --- | --- |
| *CD36* | NM_001285578.1 | F-1243 | TTTGGCTTAATGAGACTGGTACCA | 90 | TAAAGCGGATGTTCAGAAGCAAGTGACGGGGAAAATAAACCTCCTTGGCCTGGA |
| *.* | . | R-1332 | CCAGGCCAAGGAGGTTTATTT |  |  |
| *FABP3* | NM_001285701.1 | F-69 | CACTCGGTGTCGGTTTTGCT | 120 | TTGACAGCCTACCACAATCATCGAAGTGAATGGGGACACAGTCATCATAAAAACACAAAGCACCTTCAAGAACACAGAGAA |
| *.* | . | R-188 | TCTCTGTGTTCTTGAAGGTGCTTT |  |  |
| *INSR* | XM_018051134.1 | F-751 | CAGAAAGTGTGTCCGACTATCTGTAAG | 199 | ATGGGCTGCACTCTGAGGGTCTCTGCTGCCACAGCGAGTGCTTGGGCAACTGCTCAGAGCCCGACGACCCCACCAAGTGCGTGGCCTGTCGCAACTTCTACCTGGACGGCAGGTGCGTGGAGACCTGCCCGCCCCCTTACTACCACTTCCAAGACTGGCGCTGCGTGA |
| *.* | . | R-949 | TCACGCAGCGCCAGTCT |  |  |
| *IRS1* | XM_018058864.1 | F-1475 | TCCATCCCCATGCCTTCTT | 100 | GCTTCGCAAGCCCCGTCAGTCTGTCGTCCAGCAGCACGAGTGGCCACGGCTCCACCTCAGACTGCCA |
| *.* | . | R-1574 | GGCAGTCTGAGGTGGAGCC |  |  |
| *KRT8* | XM_005679931.1 | F-612 | GGCTGGTGGAGGACTTCAAG | 105 | GACTAGGATCACAGCGCACAGACATGGAGAATGAATTTGTCATCATCAAGAAGGATGTGGATAGAAGCTTACAAAA |
| *.* | . | R-716 | TGTAAGCTTCATCCACATCCTTCTT |  |  |
| *NFE2L2* | NM_001314327.1 | F-419 | CATTCCCAAAGCAGATGATTTG | 91 | TATGCATGCAGCTTTTGGCAGAGACATTCCCGTTTGTAGATGACAATGAGGTTTCTTCAGA |
| *.* | . | R-509 | CGAAGAAACCTCATTGTCATCTACA |  |  |
| *NRF1* | XM_018046932.1 | F-4072 | CGGGCCCTTGCTAAACCT | 126 | aAgttTGgtGCcaCTGGCTttcccAGCtaaAATGATGCtcTCCttgCcACCTcCATcACCCCTTtcTtTcCTTTTGTTAGGgTTAtggA |
| *.* | . | R-4197 | CCTAACCCTAACAAAAGGAAAGAAAG |  |  |
| *SLC27A6* | XM_018050707.1 | F-1124 | ACTGAGCACAGCAACTGATTGTC | 116 | CCATGCTATCTCAACACCAAGTCTCCTTATCTTTATATTTTTACTTCTGGAACAACAGGTCTACCAAAAGCAGCTAA |
| *.* | . | R-1239 | TAGCTGCTTTTGGTAGACCTGTTGT |  |  |
| *SLC2A4* | NM_001314227.1 | F-1051 | TCAATGCGGTTTTCTATTATTCGA | 80 | TTGAGTGCAGGGTAGAGAACCAGCCTATGCCACCATCGGAGCCA |
| *.* | . | R-1130 | GGCTCCGATGGTGGCATA |  |  |
| *VLDLR* | XM_018052030.1 | F-1332 | CAGTAGGCAAAGAGCCGAGTCT | 110 | gaCGAgaatCAGGAAGAtTGGCTTGGAGAGGAAAGAATATATCCAACTAGTTGAACAGCTGCGAAACACTGTGA |
| *.* | . | R-1441 | CACAGTGTTTCGCAGCTGTTC |  |  |
