# Supplementary material for: 2,4-Thiazolidinedione in Well-Fed Lactating Dairy Goats: I. Effect on Adiposity and Milk Fat Synthesis
Source: Vet Sci. 2019 May 17;6(2):45. doi: 10.3390/vetsci6020045 (PMC6632146; doi:10.3390/vetsci6020045)
Supplement: Supplementary file 1 [file vetsci-06-00045-s001.zip › vetsci-484037-supplementary/Table S5.docx]

**Table S5.** Fatty acid profile (%) in milk of goats receiving daily intrajugular injection of 2,4-thiazolidinedione (TZD) or saline (CTR).

| **Fatty Acid%** |  | **Day of TZD injection** | | | |  | ***P-value*** | | |
| --- | --- | --- | --- | --- | --- | --- | --- | --- | --- |
|  |  | **0** | **2** | **5** | **15** | **SEM** | ***TZD*** | ***Time*** | ***Z x T*** |
| **C4:0** | **CTR** | 1.76 | 1.40 | 1.57 | 1.88 | 0.17 | *0.47* | *<0.01* | *0.80* |
|  | **TZD** | 1.76 | 1.34 | 1.77 | 2.06 |  |  |  |  |
| **C6:0** | **CTR** | 2.51 | 2.13 | 2.46 | 2.79 | 0.12 | *0.52* | *<0.01* | *0.70* |
|  | **TZD** | 2.51 | 2.38 | 2.51 | 2.72 |  |  |  |  |
| **C8:0** | **CTR** | 2.42 | 2.12 | 2.65 | 2.86 | 0.18 | *0.95* | *<0.01* | *0.99* |
|  | **TZD** | 2.42 | 2.17 | 2.61 | 2.83 |  |  |  |  |
| **C10:0** | **CTR** | 7.28 | 6.30 | 8.13 | 9.47 | 0.57 | *0.98* | *<0.01* | *0.99* |
|  | **TZD** | 7.28 | 6.37 | 8.06 | 9.52 |  |  |  |  |
| **C11:0** | **CTR** | 0.09 | 0.08 | 0.13 | 0.14 | 0.01 | *0.97* | *<0.01* | *0.47* |
|  | **TZD** | 0.09 | 0.08 | 0.11 | 0.16 |  |  |  |  |
| **C12:0** | **CTR** | 2.99 | 2.94 | 3.81 | 4.43 | 0.25 | *0.35* | *<0.01* | *0.92* |
|  | **TZD** | 2.99 | 2.84 | 3.49 | 4.18 |  |  |  |  |
| **C13:1** | **CTR** | 0.13 | 0.13 | 0.14 | 0.11 | 0.01 | *0.92* | *0.57* | *0.37* |
|  | **TZD** | 0.13 | 0.12 | 0.13 | 0.13 |  |  |  |  |
| **C14:0** | **CTR** | 9.25 | 9.28 | 10.8 | 11.0 | 0.04 | *0.59* | *<0.01* | *0.67* |
|  | **TZD** | 9.25 | 9.48 | 10.0 | 10.9 |  |  |  |  |
| **C14:1cis7** | **CTR** | 0.25 | 0.25 | 0.24 | 0.19 | 0.01 | *0.03* | *<0.01* | *0.30* |
|  | **TZD** | 0.25 | 0.27 | 0.26 | 0.24 |  |  |  |  |
| **C14:1cis9** | **CTR** | 0.41 | 0.43 | 0.45 | 0.37 | 0.03 | *0.44* | *0.39* | *0.10* |
|  | **TZD** | 0.41 | 0.38 | 0.47 | 0.49 |  |  |  |  |
| **C14:1??** | **CTR** | 0.10 | 0.07 | 0.13 | 0.14 | 0.02 | *0.62* | *0.08* | *0.89* |
|  | **TZD** | 0.10 | 0.08 | 0.11 | 0.11 |  |  |  |  |
| **C15:0** | **CTR** | 1.06 | 1.10 | 1.20 | 1.17 | 0.06 | *0.09* | *0.01* | *0.44* |
|  | **TZD** | 1.06 | 1.18 | 1.24 | 1.36 |  |  |  |  |
| **C15:1** | **CTR** | 0.30 | 0.36 | 0.38 | 0.24 | 0.03 | *0.57* | *0.05* | *0.20* |
|  | **TZD** | 0.30 | 0.34 | 0.35 | 0.34 |  |  |  |  |
| **C16:0** | **CTR** | 25.9 | 26.1 | 27.6 | 29.2 | 1.11 | *0.56* | *<0.01* | *0.76* |
|  | **TZD** | 25.9 | 25.4 | 25.9 | 29.7 |  |  |  |  |
| **C16:1cis9** | **CTR** | 0.53 | 0.56 | 0.52 | 0.38 | 0.03 | *0.05* | *<0.01* | *0.42* |
|  | **TZD** | 0.53 | 0.60 | 0.55 | 0.48 |  |  |  |  |
| **C16:1cis?** | **CTR** | 0.53 | 0.68 | 0.58 | 0.58 | 0.05 | *0.73* | *0.13* | *0.98* |
|  | **TZD** | 0.53 | 0.67 | 0.58 | 0.54 |  |  |  |  |
| **C16:1trans?** | **CTR** | 0.66 | 0.70 | 0.59 | 0.40 | 0.03 | *<0.01* | *<0.01* | *0.34* |
|  | **TZD** | 0.66 | 0.75 | 0.70 | 0.50 |  |  |  |  |
| **C17:0anteiso** | **CTR** | 0.32 | 0.36 | 0.34 | 0.32 | 0.02 | *0.76* | *0.10* | *0.84* |
|  | **TZD** | 0.32 | 0.34 | 0.37 | 0.32 |  |  |  |  |
| **C17:1cis12** | **CTR** | 0.34 | 0.44 | 0.35 | 0.25 | 0.04 | *0.99* | *<0.01* | *0.92* |
|  | **TZD** | 0.34 | 0.44 | 0.38 | 0.22 |  |  |  |  |
| **C18:0** | **CTR** | 12.6 | 12.1 | 9.62 | 8.01 | 0.75 | *0.06* | *<0.01* | *0.72* |
|  | **TZD** | 12.6 | 13.8 | 11.0 | 9.18 |  |  |  |  |
| **C18:0iso** | **CTR** | 0.95 | 1.06 | 0.95 | 0.77 | 0.04 | *0.55* | *<0.01* | *0.52* |
|  | **TZD** | 0.95 | 1.01 | 0.99 | 0.85 |  |  |  |  |
| **C18:1cis?** | **CTR** | 0.14 | 0.13 | 0.15 | 0.17 | 0.02 | *0.19* | *0.54* | *0.21* |
|  | **TZD** | 0.14 | 0.14 | 0.11 | 0.13 |  |  |  |  |
| **C18:1cis9** | **CTR** | 21.1 | 23.8 | 18.7 | 17.8 | 1.32 | *0.67* | *<0.01* | *0.51* |
|  | **TZD** | 21.1 | 22.5 | 20.4 | 15.7 |  |  |  |  |
| **C18:1cis11** | **CTR** | 0.51 | 0.57 | 0.47 | 0.47 | 0.04 | *0.51* | *0.01* | *0.48* |
|  | **TZD** | 0.51 | 0.58 | 0.48 | 0.37 |  |  |  |  |
| **C18:1cis12** | **CTR** | 0.12 | 0.13 | 0.10 | 0.15 | 0.15 | *<0.01* | *0.07* | *0.23* |
|  | **TZD** | 0.12 | 0.10 | 0.07 | 0.08 |  |  |  |  |
| **C18:1cis14** | **CTR** | 0.25 | 0.22 | 0.20 | 0.28 | 0.03 | *0.06* | *0.03* | *0.37* |
|  | **TZD** | 0.25 | 0.22 | 0.14 | 0.20 |  |  |  |  |
| **C18:1tr11** | **CTR** | 0.25 | 0.29 | 0.26 | 0.23 | 0.02 | *0.58* | *0.03* | *0.76* |
|  | **TZD** | 0.25 | 0.33 | 0.24 | 0.25 |  |  |  |  |
| **C18:1tr12** | **CTR** | 1.65 | 1.43 | 1.41 | 1.11 | 0.16 | *0.11* | *0.18* | *0.69* |
|  | **TZD** | 1.65 | 1.66 | 1.58 | 1.49 |  |  |  |  |
| **C18:1tr13** | **CTR** | 0.14 | 0.16 | 0.12 | 0.16 | 0.03 | *0.53* | *0.68* | *0.90* |
|  | **TZD** | 0.14 | 0.13 | 0.12 | 0.15 |  |  |  |  |
| **C18:1tr16** | **CTR** | 0.13 | 0.12 | 0.11 | 0.03 | 0.01 | *0.22* | *<0.01* | *0.08* |
|  | **TZD** | 0.13 | 0.12 | 0.10 | 0.08 |  |  |  |  |
| **C18:2cis9cis12** | **CTR** | 2.05 | 2.17 | 1.95 | 1.67 | 0.13 | *0.65* | *<0.01* | *0.95* |
|  | **TZD** | 2.05 | 2.03 | 1.93 | 1.65 |  |  |  |  |
| **C18:2cis9tr12** | **CTR** | 0.10 | 0.09 | 0.12 | 0.12 | 0.02 | *0.88* | *0.63* | *0.94* |
|  | **TZD** | 0.10 | 0.10 | 0.11 | 0.10 |  |  |  |  |
| **C18:3n3** | **CTR** | 0.85 | 0.89 | 0.92 | 0.77 | 0.08 | *0.68* | *0.09* | *0.87* |
|  | **TZD** | 0.85 | 0.91 | 0.91 | 0.66 |  |  |  |  |
| **C18:3tr9cis12cis15** | **CTR** | 0.07 | 0.07 | 0.03 | 0.04 | 0.01 | *0.04* | *<0.01* | *0.49* |
|  | **TZD** | 0.07 | 0.10 | 0.06 | 0.05 |  |  |  |  |
| **C18:3CLA** | **CTR** | 0.63 | 0.49 | 0.51 | 0.58 | 0.10 | *0.45* | *0.23* | *0.92* |
|  | **TZD** | 0.63 | 0.35 | 0.46 | 0.56 |  |  |  |  |
| **C18:3tr9tr2tr5** | **CTR** | 0.30 | 0.27 | 0.24 | 0.21 | 0.02 | *<0.01* | *<0.01* | *0.25* |
|  | **TZD** | 0.30 | 0.30 | 0.28 | 0.28 |  |  |  |  |
| **C19:1** | **CTR** | 0.12 | 0.14 | 0.16 | 0.14 | 0.01 | *0.03* | *0.40* | *0.57* |
|  | **TZD** | 0.12 | 0.11 | 0.12 | 0.11 |  |  |  |  |
| **C20:2tr** | **CTR** | 0.09 | 0.18 | 0.12 | 0.11 | 0.03 | *0.33* | *0.07* | *0.86* |
|  | **TZD** | 0.09 | 0.14 | 0.12 | 0.08 |  |  |  |  |
| **C20:4n6** | **CTR** | 0.15 | 0.16 | 0.15 | 0.12 | 0.01 | *0.25* | *0.30* | *0.07* |
|  | **TZD** | 0.15 | 0.14 | 0.17 | 0.16 |  |  |  |  |
| **C20:5n3** | **CTR** | 0.15 | 0.10 | 0.16 | 0.12 | 0.02 | *0.15* | *0.14* | *0.54* |
|  | **TZD** | 0.15 | 0.08 | 0.10 | 0.09 |  |  |  |  |
| **C22:0** | **CTR** | 0.11 | 0.10 | 0.10 | 0.09 | 0.01 | *<0.01* | *0.93* | *0.25* |
|  | **TZD** | 0.11 | 0.13 | 0.12 | 0.13 |  |  |  |  |
| **C22:5n3** | **CTR** | 0.45 | 0.30 | 0.37 | 0.42 | 0.06 | *0.56* | *0.34* | *0.89* |
|  | **TZD** | 0.45 | 0.37 | 0.43 | 0.40 |  |  |  |  |
| **C26:0** | **CTR** | 0.08 | 0.11 | 0.12 | 0.10 | 0.02 | *0.51* | *0.55* | *0.87* |
|  | **TZD** | 0.08 | 0.11 | 0.09 | 0.09 |  |  |  |  |
| **Denovo** | **CTR** | 40.8 | 38.8 | 45.5 | 48.3 | 1.60 | *0.57* | *<0.01* | *0.83* |
|  | **TZD** | 40.8 | 38.1 | 43.2 | 48.7 |  |  |  |  |
| **Preformed** | **CTR** | 59.2 | 61.2 | 54.4 | 51.7 | 1.61 | *0.57* | *<0.01* | *0.83* |
|  | **TZD** | 59.2 | 61.9 | 56.8 | 51.3 |  |  |  |  |
| **Saturated** | **CTR** | 66.0 | 63.8 | 68.9 | 71.2 | 1.69 | *0.98* | *<0.01* | *0.75* |
|  | **TZD** | 66.0 | 63.3 | 66.9 | 72.8 |  |  |  |  |
| **Unsaturated** | **CTR** | 33.8 | 36.0 | 31.0 | 28.3 | 1.70 | *0.95* | *<0.01* | *0.79* |
|  | **TZD** | 33.8 | 35.6 | 33.0 | 27.1 |  |  |  |  |
| **Δ9 C14^1^** | **CTR** | 0.46 | 0.53 | 0.41 | 0.39 | 0.04 | *0.02* | *0.02* | *0.32* |
|  | **TZD** | 0.46 | 0.61 | 0.57 | 0.45 |  |  |  |  |
| **Δ9 C16^1^** | **CTR** | 0.02 | 0.02 | 0.02 | 0.01 | 0.002 | *0.42* | *<0.01* | *0.69* |
|  | **TZD** | 0.02 | 0.02 | 0.02 | 0.02 |  |  |  |  |
| **Δ9 C18^1^** | **CTR** | 0.63 | 0.66 | 0.66 | 0.69 | 0.02 | *0.05* | *0.09* | *0.42* |
|  | **TZD** | 0.63 | 0.62 | 0.65 | 0.64 |  |  |  |  |
| **Δ9 C18 tr12^1^** | **CTR** | 0.44 | 0.39 | 0.57 | 0.40 | 0.05 | *0.39* | *0.02* | *0.56* |
|  | **TZD** | 0.44 | 0.44 | 0.54 | 0.49 |  |  |  |  |
| **Δ9 desaturation^1^** | **CTR** | 0.31 | 0.34 | 0.29 | 0.27 | 0.02 | *0.67* | *<0.01* | *0.48* |
|  | **TZD** | 0.31 | 0.32 | 0.31 | 0.25 |  |  |  |  |

^1^Delta 9 desaturase indexes were calculated as follow:

- Δ9 C14 = cis9 C14:1/(C14:0 + cis9 C14:1)
- Δ9 C16 = cis9 C16:1/(C16:0 + cis9 C16:1)
- Δ9 C18 = cis9 C18:1/(C18:0 + cis9 C18:1)
- Δ9 C18:1 = cis9 trans12 C18:2/(trans12 C18:1 + cis9 trans12 C18:2)
- Δ9 desaturation = sum of C14, C16, C18, trans12 C18:1 cis9/(sum of C14:0, C16:0, C18:0, trans12 C18:1 + sum of C14, C16, C18, trans12 C18:1 cis9)
