# Supplementary material for: 2,4-Thiazolidinedione in Well-Fed Lactating Dairy Goats: I. Effect on Adiposity and Milk Fat Synthesis
Source: Vet Sci. 2019 May 17;6(2):45. doi: 10.3390/vetsci6020045 (PMC6632146; doi:10.3390/vetsci6020045)
Supplement: Supplementary file 1 [file vetsci-06-00045-s001.zip › vetsci-484037-supplementary/Table S6.docx]

**Table S6.** Grams/milking of each fatty acid in milk of goats receiving daily intrajugular injection of 2,4-thiazolidinedione (TZD) or saline (CTR).

| **Fatty Acid** | **Treatment** | **Day of TZD injection** | | | |  | ***P-value*** | | |
| --- | --- | --- | --- | --- | --- | --- | --- | --- | --- |
|  |  | **0** | **2** | **5** | **15** | **SEM** | ***TZD*** | ***Time*** | ***Z × T*** |
| **C4:0** | **CTR** | 1.38 | 1.23 | 1.13 | 1.71 | 0.19 | *0.36* | *0.14* | *0.65* |
|  | **TZD** | 1.38 | 0.96 | 1.22 | 1.37 |  |  |  |  |
| **C6:0** | **CTR** | 1.70 | 1.55 | 1.43 | 1.90 | 0.22 | *0.27* | *0.30* | *0.84* |
|  | **TZD** | 1.70 | 1.30 | 1.35 | 1.54 |  |  |  |  |
| **C8:0** | **CTR** | 1.79 | 1.66 | 1.62 | 2.08 | 0.25 | *0.37* | *0.41* | *0.93* |
|  | **TZD** | 1.79 | 1.45 | 1.49 | 1.76 |  |  |  |  |
| **C10:0** | **CTR** | 6.77 | 6.23 | 6.59 | 8.54 | 0.94 | *0.50* | *0.17* | *0.96* |
|  | **TZD** | 6.77 | 5.90 | 5.98 | 7.62 |  |  |  |  |
| **C12:0** | **CTR** | 3.25 | 3.29 | 3.36 | 4.23 | 0.45 | *0.48* | *0.19* | *0.95* |
|  | **TZD** | 3.25 | 3.17 | 2.93 | 3.84 |  |  |  |  |
| **C13:1** | **CTR** | 0.07 | 0.07 | 0.06 | 0.04 | 0.02 | *0.52* | *0.29* | *0.61* |
|  | **TZD** | 0.08 | 0.04 | 0.04 | 0.05 |  |  |  |  |
| **C14:0** | **CTR** | 10.8 | 11.2 | 10.7 | 12.6 | 1.27 | *0.42* | *0.57* | *0.91* |
|  | **TZD** | 10.8 | 11.0 | 9.5 | 11.1 |  |  |  |  |
| **C14:1cis7** | **CTR** | 0.25 | 0.26 | 0.21 | 0.20 | 0.03 | *0.90* | *0.21* | *0.99* |
|  | **TZD** | 0.25 | 0.26 | 0.21 | 0.20 |  |  |  |  |
| **C14:1cis9** | **CTR** | 0.38 | 0.35 | 0.36 | 0.37 | 0.06 | *0.95* | *0.83* | *0.97* |
|  | **TZD** | 0.38 | 0.34 | 0.34 | 0.40 |  |  |  |  |
| **C14:1??** | **CTR** | 0.08 | 0.06 | 0.08 | 0.11 | 0.03 | *0.91* | *0.76* | *0.31* |
|  | **TZD** | 0.08 | 0.13 | 0.06 | 0.07 |  |  |  |  |
| **C15:0** | **CTR** | 0.96 | 1.09 | 0.93 | 1.02 | 0.11 | *0.82* | *0.43* | *0.99* |
|  | **TZD** | 0.96 | 1.13 | 0.92 | 1.07 |  |  |  |  |
| **C15:1** | **CTR** | 0.37 | 0.44 | 0.39 | 0.34 | 0.05 | *0.20* | *0.93* | *0.54* |
|  | **TZD** | 0.37 | 0.30 | 0.34 | 0.34 |  |  |  |  |
| **C16:0** | **CTR** | 19.8 | 20.5 | 18.1 | 22.4 | 2.07 | *0.35* | *0.33* | *0.88* |
|  | **TZD** | 19.8 | 19.8 | 16.3 | 19.1 |  |  |  |  |
| **C16:1cis9** | **CTR** | 0.58 | 0.69 | 0.53 | 0.50 | 0.07 | *0.46* | *0.11* | *0.96* |
|  | **TZD** | 0.58 | 0.61 | 0.50 | 0.47 |  |  |  |  |
| **C16:1cis?** | **CTR** | 0.62 | 0.48 | 0.61 | 0.68 | 0.10 | *0.75* | *0.88* | *0.68* |
|  | **TZD** | 0.62 | 0.59 | 0.55 | 0.53 |  |  |  |  |
| **C16:1trans?** | **CTR** | 0.71 | 0.77 | 0.61 | 0.55 | 0.07 | *0.82* | *0.03* | *0.99* |
|  | **TZD** | 0.71 | 0.75 | 0.61 | 0.52 |  |  |  |  |
| **C17:0** | **CTR** | 0.32 | 0.37 | 0.32 | 0.33 | 0.08 | *0.94* | *<0.01* | *0.65* |
|  | **TZD** | 0.32 | 0.38 | 0.34 | 0.29 |  |  |  |  |
| **C17:0anteiso** | **CTR** | 0.40 | 0.44 | 0.39 | 0.42 | 0.04 | *0.39* | *0.65* | *0.92* |
|  | **TZD** | 0.40 | 0.42 | 0.37 | 0.36 |  |  |  |  |
| **C17:1cis12** | **CTR** | 0.56 | 0.67 | 0.55 | 0.52 | 0.07 | *0.31* | *0.13* | *0.83* |
|  | **TZD** | 0.56 | 0.61 | 0.52 | 0.39 |  |  |  |  |
| **C17:1cis10** | **CTR** | 0.19 | 0.26 | 0.18 | 0.04 | 0.04 | *0.37* | *<0.01* | *0.86* |
|  | **TZD** | 0.20 | 0.25 | 0.23 | 0.10 |  |  |  |  |
| **C18:0** | **CTR** | 12.8 | 12.8 | 9.8 | 10.1 | 1.52 | *0.52* | *0.03* | *0.91* |
|  | **TZD** | 12.8 | 12.7 | 9.0 | 8.1 |  |  |  |  |
| **C18:0iso** | **CTR** | 1.13 | 1.24 | 1.04 | 1.04 | 0.13 | *0.34* | *0.31* | *0.92* |
|  | **TZD** | 1.13 | 1.13 | 0.97 | 0.87 |  |  |  |  |
| **C18:1cis9** | **CTR** | 19.1 | 21.5 | 16.3 | 17.8 | 2.09 | *0.13* | *0.06* | *0.60* |
|  | **TZD** | 19.1 | 18.7 | 14.9 | 12.3 |  |  |  |  |
| **C18:1cis11** | **CTR** | 0.67 | 0.45 | 0.63 | 0.67 | 0.09 | *0.75* | *0.46* | *0.62* |
|  | **TZD** | 0.67 | 0.56 | 0.57 | 0.52 |  |  |  |  |
| **C18:1cis12** | **CTR** | 0.32 | 0.35 | 0.28 | 0.37 | 0.03 | *0.27* | *0.28* | *0.63* |
|  | **TZD** | 0.32 | 0.33 | 0.27 | 0.29 |  |  |  |  |
| **C18:1cis14** | **CTR** | 0.44 | 0.44 | 0.41 | 0.48 | 0.04 | *0.15* | *0.36* | *0.71* |
|  | **TZD** | 0.44 | 0.42 | 0.33 | 0.38 |  |  |  |  |
| **C18:1*cis*isomer** | **CTR** | 0.33 | 0.20 | 0.33 | 0.36 | 0.04 | *0.88* | *0.17* | *0.56* |
|  | **TZD** | 0.33 | 0.27 | 0.29 | 0.31 |  |  |  |  |
| **C18:1tr11** | **CTR** | 0.43 | 0.49 | 0.42 | 0.44 | 0.04 | *0.74* | *0.13* | *0.71* |
|  | **TZD** | 0.43 | 0.54 | 0.38 | 0.39 |  |  |  |  |
| **C18:1tr12** | **CTR** | 1.56 | 1.15 | 1.28 | 1.26 | 0.23 | *0.75* | *0.50* | *0.92* |
|  | **TZD** | 1.56 | 1.43 | 1.28 | 1.20 |  |  |  |  |
| **C18:1tr13** | **CTR** | 0.30 | 0.37 | 0.29 | 0.33 | 0.04 | *0.39* | *0.82* | *0.81* |
|  | **TZD** | 0.30 | 0.28 | 0.28 | 0.31 |  |  |  |  |
| **C18:1tr16** | **CTR** | 0.30 | 0.31 | 0.26 | 0.08 | 0.04 | *0.14* | *<0.01* | *0.17* |
|  | **TZD** | 0.30 | 0.31 | 0.27 | 0.24 |  |  |  |  |
| **C18:2cis9cis12** | **CTR** | 2.25 | 2.40 | 2.04 | 2.08 | 0.22 | *0.11* | *0.16* | *0.79* |
|  | **TZD** | 2.25 | 2.08 | 1.74 | 1.64 |  |  |  |  |
| **C18:2cis9tr12** | **CTR** | 0.27 | 0.28 | 0.28 | 0.29 | 0.03 | *0.89* | *0.96* | *0.95* |
|  | **TZD** | 0.27 | 0.29 | 0.27 | 0.27 |  |  |  |  |
| **C18:3n3** | **CTR** | 1.03 | 1.09 | 1.01 | 1.01 | 0.12 | *0.36* | *0.76* | *0.93* |
|  | **TZD** | 1.03 | 1.02 | 0.92 | 0.86 |  |  |  |  |
| **C18:3tr9cis12cis15** | **CTR** | 0.19 | 0.22 | 0.10 | 0.15 | 0.05 | *0.34* | *0.09* | *0.80* |
|  | **TZD** | 0.19 | 0.31 | 0.15 | 0.15 |  |  |  |  |
| **C18:3tr9tr2tr15** | **CTR** | 0.43 | 0.44 | 0.38 | 0.34 | 0.04 | *0.50* | *0.23* | *0.91* |
|  | **TZD** | 0.43 | 0.47 | 0.38 | 0.40 |  |  |  |  |
| **C18:3CLA** | **CTR** | 0.61 | 0.59 | 0.54 | 0.64 | 0.07 | *0.19* | *0.42* | *0.77* |
|  | **TZD** | 0.61 | 0.45 | 0.51 | 0.55 |  |  |  |  |
| **C19:1** | **CTR** | 0.30 | 0.33 | 0.32 | 0.33 | 0.03 | *0.67* | *0.64* | *0.80* |
|  | **TZD** | 0.30 | 0.36 | 0.30 | 0.29 |  |  |  |  |
| **C20:2tr** | **CTR** | 0.27 | 0.35 | 0.29 | 0.29 | 0.05 | *0.53* | *0.17* | *0.83* |
|  | **TZD** | 0.27 | 0.37 | 0.23 | 0.24 |  |  |  |  |
| **C20:4n6** | **CTR** | 0.33 | 0.36 | 0.33 | 0.29 | 0.03 | *0.70* | *0.72* | *0.86* |
|  | **TZD** | 0.33 | 0.35 | 0.33 | 0.33 |  |  |  |  |
| **C20:5n3** | **CTR** | 0.36 | 0.35 | 0.37 | 0.32 | 0.05 | *0.80* | *0.90* | *0.92* |
|  | **TZD** | 0.36 | 0.36 | 0.32 | 0.33 |  |  |  |  |
| **C22:0** | **CTR** | 0.29 | 0.31 | 0.29 | 0.22 | 0.04 | *0.10* | *0.54* | *0.54* |
|  | **TZD** | 0.29 | 0.37 | 0.31 | 0.33 |  |  |  |  |
| **C22:5n3** | **CTR** | 0.62 | 0.54 | 0.53 | 0.77 | 0.09 | *0.50* | *0.52* | *0.47* |
|  | **TZD** | 0.62 | 0.58 | 0.54 | 0.54 |  |  |  |  |
| **C26:0** | **CTR** | 0.17 | 0.26 | 0.24 | 0.55 | 0.10 | *0.18* | *0.35* | *0.19* |
|  | **TZD** | 0.19 | 0.27 | 0.18 | 0.16 |  |  |  |  |
| **De novo** | **CTR** | 37.3 | 37.0 | 35.5 | 43.8 | 4.30 | *0.40* | *0.40* | *0.93* |
|  | **TZD** | 37.3 | 35.4 | 32.0 | 38.2 |  |  |  |  |
| **Preformed** | **CTR** | 58.4 | 61.6 | 50.6 | 55.2 | 5.96 | *0.29* | *0.16* | *0.82* |
|  | **TZD** | 58.4 | 58.2 | 46.5 | 44.0 |  |  |  |  |
| **Saturated** | **CTR** | 59.8 | 60.0 | 54.3 | 65.4 | 6.58 | *0.40* | *0.49* | *0.90* |
|  | **TZD** | 59.8 | 58.1 | 49.1 | 56.0 |  |  |  |  |
| **Unsaturated** | **CTR** | 35.8 | 38.4 | 31.5 | 33.1 | 3.67 | *0.25* | *0.15* | *0.81* |
|  | **TZD** | 35.8 | 35.3 | 29.2 | 26.1 |  |  |  |  |
| **Δ9 C14** | **CTR** | 0.034 | 0.029 | 0.031 | 0.024 | 0.003 | *0.11* | *0.43* | *0.29* |
|  | **TZD** | 0.034 | 0.029 | 0.036 | 0.035 |  |  |  |  |
| **Δ9 C16** | **CTR** | 0.028 | 0.033 | 0.028 | 0.020 | 0.002 | *0.46* | *<0.01* | *0.47* |
|  | **TZD** | 0.028 | 0.031 | 0.031 | 0.024 |  |  |  |  |
| **Δ9 C18** | **CTR** | 0.61 | 0.64 | 0.64 | 0.67 | 0.02 | *0.04* | *0.10* | *0.39* |
|  | **TZD** | 0.61 | 0.60 | 0.63 | 0.62 |  |  |  |  |
| **Δ9 C18:1** | **CTR** | 0.16 | 0.29 | 0.21 | 0.23 | 0.03 | *0.27* | *0.08* | *0.83* |
|  | **TZD** | 0.16 | 0.23 | 0.20 | 0.19 |  |  |  |  |
| **Δ9 desaturation** | **CTR** | 0.31 | 0.34 | 0.29 | 0.28 | 0.02 | *0.46* | *<0.01* | *0.35* |
|  | **TZD** | 0.31 | 0.31 | 0.31 | 0.26 |  |  |  |  |
