# Supplementary figures and images for: 2,4-Thiazolidinedione in Well-Fed Lactating Dairy Goats: I. Effect on Adiposity and Milk Fat Synthesis
Source: Vet Sci. 2019 May 17;6(2):45. doi: 10.3390/vetsci6020045 (PMC6632146; doi:10.3390/vetsci6020045)

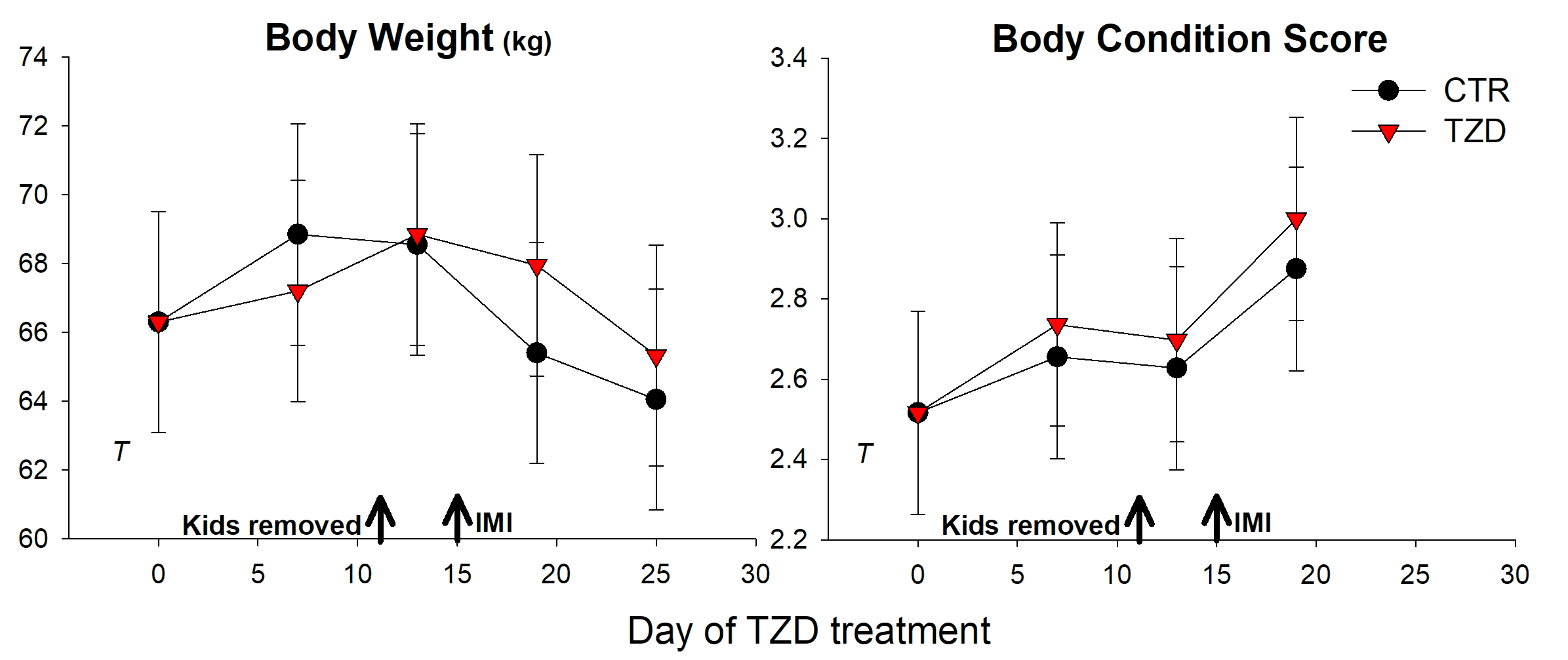

Supplement: Supplementary file 1 [file vetsci-06-00045-s001.zip › vetsci-484037-supplementary/Figure S1.TIF]

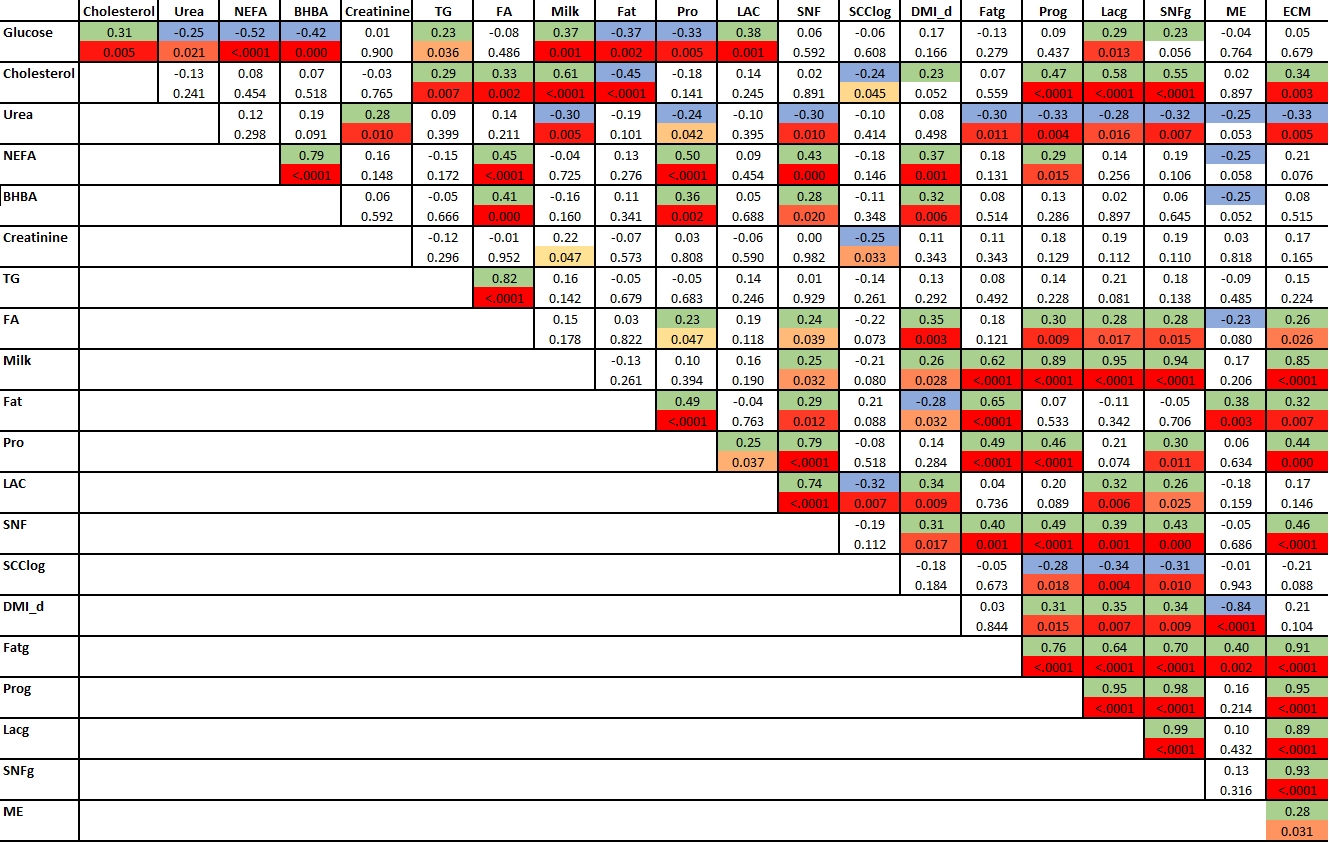

Supplement: Supplementary file 1 [file vetsci-06-00045-s001.zip › vetsci-484037-supplementary/Figure S2.TIF]

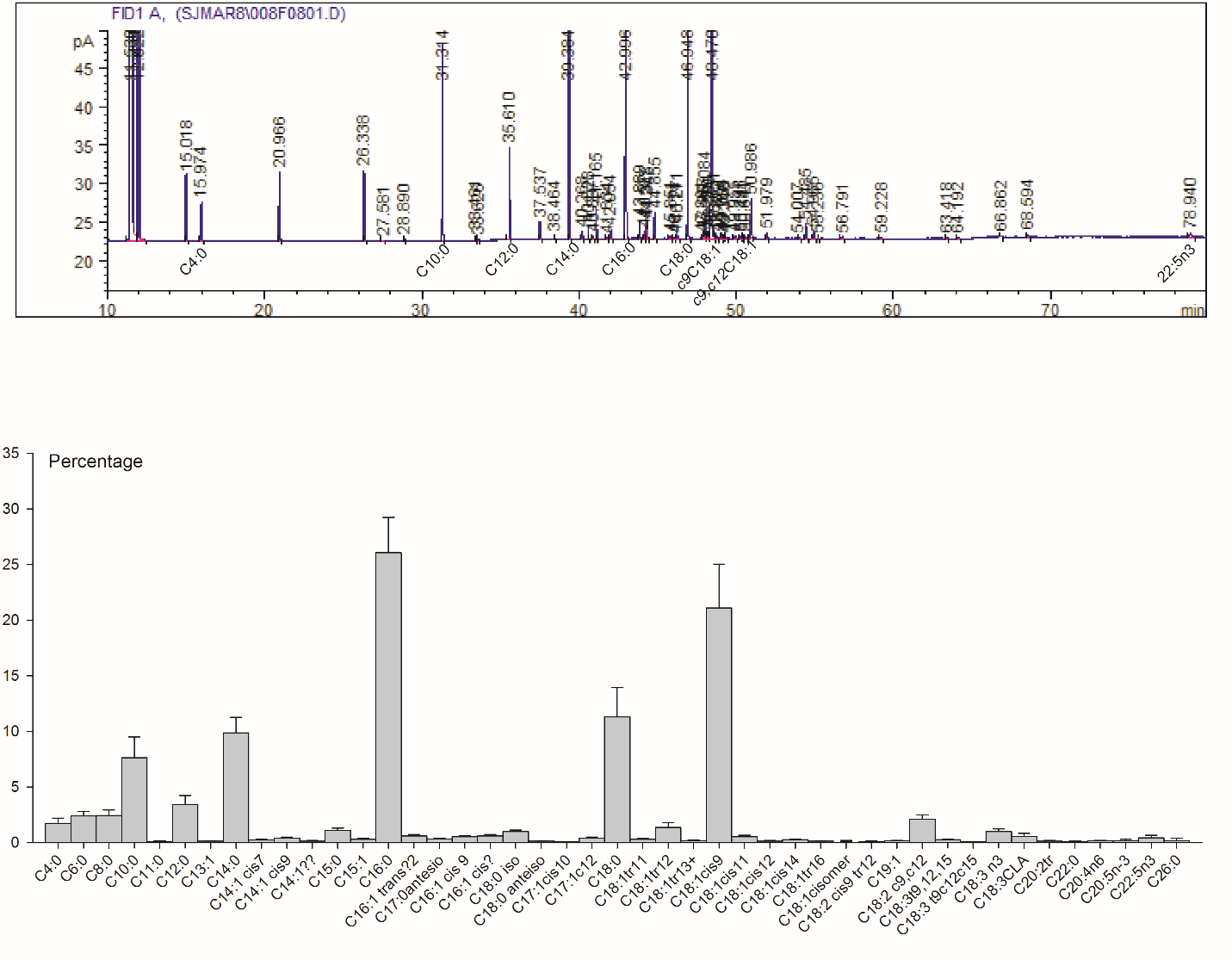

Supplement: Supplementary file 1 [file vetsci-06-00045-s001.zip › vetsci-484037-supplementary/Figure S3.TIF]
